# LISA: Accurate reconstruction of cell trajectory and pseudo-time for massive single cell RNA-seq data

**Published:** 2019

**Authors:** Yang Chen, Yuping Zhang, Zhengqing Ouyang

**Affiliations:** 1 The Jackson Laboratory for Genomic Medicine, Farmington, CT 06032, USA; 2 Department of Statistics, University of Connecticut, Storrs, CT 06269, USA; 3 Institute for Systems Genomics, University of Connecticut, Storrs, CT 06269, USA; 4 Department of Biomedical Engineering, University of Connecticut, Storrs, CT 06269, USA; 5 Department of Genetics and Genome Sciences, University of Connecticut, Farmington, CT 06030, USA; 6 Center for Quantitative Medicine, University of Connecticut Health Center, Farmington, CT 06030, USA

**Keywords:** Single cell RNA-seq, cell trajectory, pseudo-time, manifold learning

## Abstract

Cell trajectory reconstruction based on single cell RNA sequencing is important for obtaining the landscape of different cell types and discovering cell fate transitions. Despite intense effort, analyzing massive single cell RNA-seq datasets is still challenging. We propose a new method named Landmark Isomap for Single-cell Analysis (LISA). LISA is an unsupervised approach to build cell trajectory and compute pseudo-time in the isometric embedding based on geodesic distances. The advantages of LISA include: (1) It utilizes k-nearest-neighbor graph and hierarchical clustering to identify cell clusters, peaks and valleys in low-dimension representation of the data; (2) Based on Landmark Isomap, it constructs the main geometric structure of cell lineages; (3) It projects cells to the edges of the main cell trajectory to generate the global pseudo-time. Assessments on simulated and real datasets demonstrate the advantages of LISA on cell trajectory and pseudo-time reconstruction compared to Monocle2 and TSCAN. LISA is accurate, fast, and requires less memory usage, allowing its applications to massive single cell datasets generated from current experimental platforms.

## 1. Introduction

Single cell RNA sequencing (scRNA-seq) is emerging to revolutionize the study of development and disease processes. It has been widely used to investigate the dynamic gene expression landscape, cell type identification, cell state transition, and pseudo-time estimation at single cell level [1–7].

An important computational issue of scRNA-seq analysis is on the reconstruction of cell trajectory and pseudo-time for individual cells. Among existing methods, Monocle2 [8], TSCAN [9], and Slingshot [10] are shown to have relatively better performance [4]. Monocle2 utilizes the principal component analysis (PCA) and discriminative dimensionality reduction tree (DDRTree) [11]. It is often able to build a tree structure. But an arbitrarily large cell cluster number (usually > 100) is used for minimum spanning tree (MST) construction. Slingshot extends the principle curve method to fit the lineages built on MST. Similar to DDRTree, it makes the tree structure smoother. But the users need to determine the dimension reduction and clustering methods and generate cell lineages before using Slingshot. TSCAN uses Gaussian mixture models and the Bayesian information criterion for automatically determining cell cluster number, and then builds cell lineages by MST on cluster centers in the PCA space. TSCAN and Slingshot can only infer cell orders in each cell lineage and are not able to estimate the global pseudo-time of all cells. Most of the existing methods were only applied to small scRNA-seq datasets. It is not clear whether they are feasible for massive scRNA-seq datasets.

Large scale scRNA-seq technologies [5], such as 10x Genomics [12], make it possible to profile more than tens or hundreds of thousands of cells. Such massive scRNA-seq datasets promote the development of new cell trajectory reconstruction methods [1–4]. Existing literature has used empirical approaches to study cell lineages supervised by known time labels and cell marker genes [1–4]. It is not known how well one can reconstruct complex cell trajectory and pseudo-time by unsupervised approaches.

We have developed the Landmark Isomap for Single-cell Analysis (LISA), an unsupervised method aiming to reconstruct cell trajectory and pseudo-time for massive scRNA-seq datasets. Briefly, LISA first automatically determines cell clusters, peaks and valleys based on k-nearest-neighbor graph (kNN-graph) [13] and hierarchical clustering. Then it maps cells into the isometric embedding based on geodesic distances [14] using the peaks and valleys as landmarks. It then builds the MST on the cluster centers as the main cell trajectory in the isometric embedding. Finally, it computes the pseudo-time by projecting cells onto the MST.

The rest of the paper is organized as follows: in Methods, we introduce the algorithm of LISA. In Results, we assess LISA on a simulated dataset, and two large scRNA-seq datasets. One dataset is on human embryo development containing 1,364 cells [15]. The other is on zebrafish embryogenesis including 38,731 cells [2]. We compared LISA with Monocle2 and TSCAN on cell trajectory reconstruction. We also compared LISA with Monocle2 on global pseudo-time estimation. The paper is concluded with a discussion.

## 2. Methods

**Figure 1.**
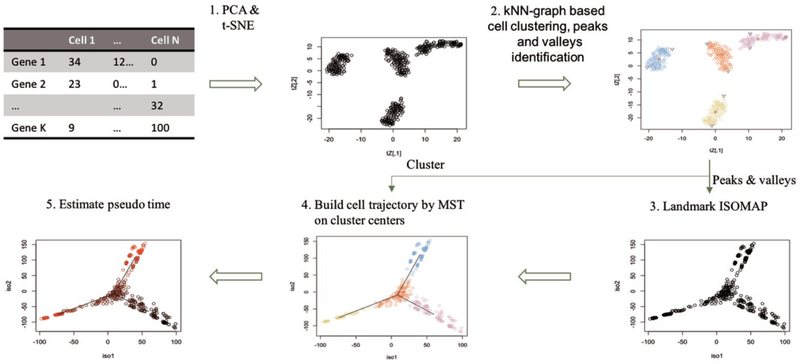
Workflow of LISA. (1) Do PCA for the gene expression matrix (K genes * N cells) and select top ranked PCs. Then the N cells with the selected PCs are mapped into the t-SNE embedding. (2) Estimate cell density in the t-SNE embedding and build the k-NN graph to find peaks and valleys. Then perform hierarchical clustering until each cluster contains one peak point (star shape). Valley points are shown as inverted triangles. (3) Using peaks and valleys as landmark points and map the N cells with the selected PCs into the isometric embedding based on geodesic distances. (4) Build the main cell trajectory using MST on the cluster centers in the isometric embedding. (5) Estimate global pseudo-time by projecting cells onto the main cell trajectory.

The workflow of LISA is shown in Fig. 1. We can start with either unnormalized or normalized gene expression values for K genes and N cells. If the input data are raw read counts, they will be log2-transformed and filtered. The filtering process removes lowly expressed genes. Usually, genes with low variances will be removed too. The details of the LISA method will be introduced as follows.

### 2.1. Visualize cells by PCA and t-Distributed Stochastic Neighbor Embedding (t-SNE)

PCA and t-SNE are two common dimensionality reduction methods for visualization. We use PCA to select top ranked PCs that keep the major variations in the data. We then derive the t-SNE [16] coordinates based on the selected PCs.

### 2.2. Identify cell clusters, peaks, and valleys

We identify cell clusters, peaks, and valleys based on kNN-graph and hierarchical clustering. We construct the kNN-graph based on the Euclidean distance with a default k as 50. To improve the speed, we use the kd-tree [13] to construct the kNN-graph, resulting a running time of O(NlogN), where N is the number of cells.

After building the kNN-graph, we then search for cell peaks and valleys. We first estimate cell density based on a nonparametric density estimation approach [17]. For each cell, if its density value is higher than all the k−1 nearest neighbors, it is regarded as a peak. Conversely, if its density value is lower than all the k−1 nearest neighbors, it is determined as a valley. Then we propose an iterative hierarchical clustering method as follows:

1. Do hierarchical clustering in the t-SNE embedding. Cut the resulting dendrogram so that the number of clusters is equal to the number of peaks.
2. Among the resulting clusters, if one cluster contains more than one peak, perform hierarchical clustering again on this cluster with the cluster number equal to the peak number in it.
3. Do step 2 until each cluster contains at most one peak.
4. For a cluster without a peak, merge it with another cluster containing a nearest peak. The nearest peak is defined as the one that is closest to the cluster with the minimum distance to the cluster.

### 2.3. Landmark Isomap

We employ the nonlinear dimension reduction method Landmark Isomap for deriving cell landscapes which preserve the geometric features of the input data. Isometric feature mapping (Isomap) [18] is based on neighborhood graph construction and multidimensional scaling of geodesic distances, with time complexity of O(N^3^). To improve the computing efficiency, we adapt the Landmark Isomap [14] to make it suitable for massive scRNA-seq datasets. When using n landmark points (n « N), it has a time complexity of O(mnNlogN) + O(n^2^N), where m is the number of the nearest neighbors for constructing the neighborhood graph. Here, we use the peaks and valleys as landmark points.

### 2.4. Estimating pseudo-time

We build the main cell trajectory by MST on the cluster centers in the isometric embedding. We then map the cells on the main cell trajectory to estimate the pseudo-time for each cell. The detailed steps are as the following:

1. Set a root node in the MST.
2. Project cells c_k_ on its nearest edge in the MST. Assume the nearest edge is $\vec{{\text{e}}_{\text{i,j}}}$ = <v_i_, v_j_>, v_i_ is closer to the root than v_j_ does. The projection vector $\vec{{\text{v}}_{\text{i}}{\text{c}}_{\text{k}}^{\prime }}$ on the vector $\vec{{\text{e}}_{\text{i,j}}}$ can be expressed as $\vec{{\text{v}}_{\text{i}}{\text{c}}_{\text{k}}^{\prime }}\;=\;\frac{\vec{{\text{v}}_{\text{i}}{\text{c}}_{\text{k}}}\cdot \vec{{\text{v}}_{\text{i}}{\text{v}}_{\text{j}}}}{\|{\text{v}}_{\text{i}}{\text{c}}_{\text{k}}\|\;\|{\text{v}}_{\text{i}}{\text{v}}_{\text{j}}\|}\;\frac{\|{\text{v}}_{\text{i}}{\text{c}}_{\text{k}}\|}{\|{\text{v}}_{\text{i}}{\text{v}}_{\text{j}}\|}\;\vec{{\text{v}}_{\text{i}}{\text{v}}_{\text{j}}}$.
3. For each projection point ${\text{c}}_{\text{k}}^{\prime }$, calculate its distance to the root as the pseudo-time. The pseudotime ${\text{t}}_{{\text{c}}_{\text{k}}}\;=\;distance(root,\;{\text{v}}_{\text{i}})\;+\;\|\vec{{\text{v}}_{\text{i}}{\text{c}}_{\text{k}}}\cdot \vec{{\text{v}}_{\text{i}}{\text{v}}_{\text{j}}}\|$. Here, *distance*(*root*, v_i_) is the length of the shortest path from v_i_ to the root in MST.

This method can run in O(N) time.

## 3. Results

To demonstrate LISA’s capabilities to accurately build cell trajectory and estimated pseudo-time, we evaluated it on one simulated dataset and two real datasets. The sizes of datasets range from several hundreds to tens of thousands. All of them contain true time labels. LISA identified cell trajectory and estimated pseudo-time for all datasets. We used the Spearman correlation coefficients between the true time labels and the estimated pseudo-time to assess the performance of LISA. Furthermore, we compared our results with two other state-of-the-art tools, Monocle2 and TSCAN.

### 3.1. Datasets

SLS3279 is a simulated dataset which contains 475 cells and 48 genes [19]. The time label ranges from 1 to 5 with continuous values. It contains two terminal lineages along with time.

The EMTAB dataset contains 1,529 cells from 88 human preimplantation embryos from E3 to E7 [15]. The processed Reads Per Kilobase of transcript per Million mapped reads (RPKM) values is downloaded from EMBL-EBI (https://www.ebi.ac.uk/). Here, we obtained 1,364 cells after filtering lowly represented cells using Seurat-1.4.1 [20]. We then used the 736 high variance genes from Petropoulos *et al.* [15]. The RPKM values were log2-transformed.

We also used 38,731 cells from zebrafish embryos across 12 developmental stages between 3.3–12 hours [2]. The raw dataset was processed by URD (https://github.com/farrellja/URD). The processed data was normalized to Transcripts Per Million (TPM) values. The TPM values were then log2-transformed. There were 1,883 highly variable genes in the dataset.

We compared the performance of LISA, Monocle2, and TSCAN on cell trajectory reconstruction. We also compared the performance of LISA and Monocle2 on global pseudo-time estimation. In the latter scenario, TSCAN was not compared as it cannot generate global pseudo-time for all cells. We also compared all three methods for running time and memory usage.

### 3.2. Simulation results

**Figure 2.**
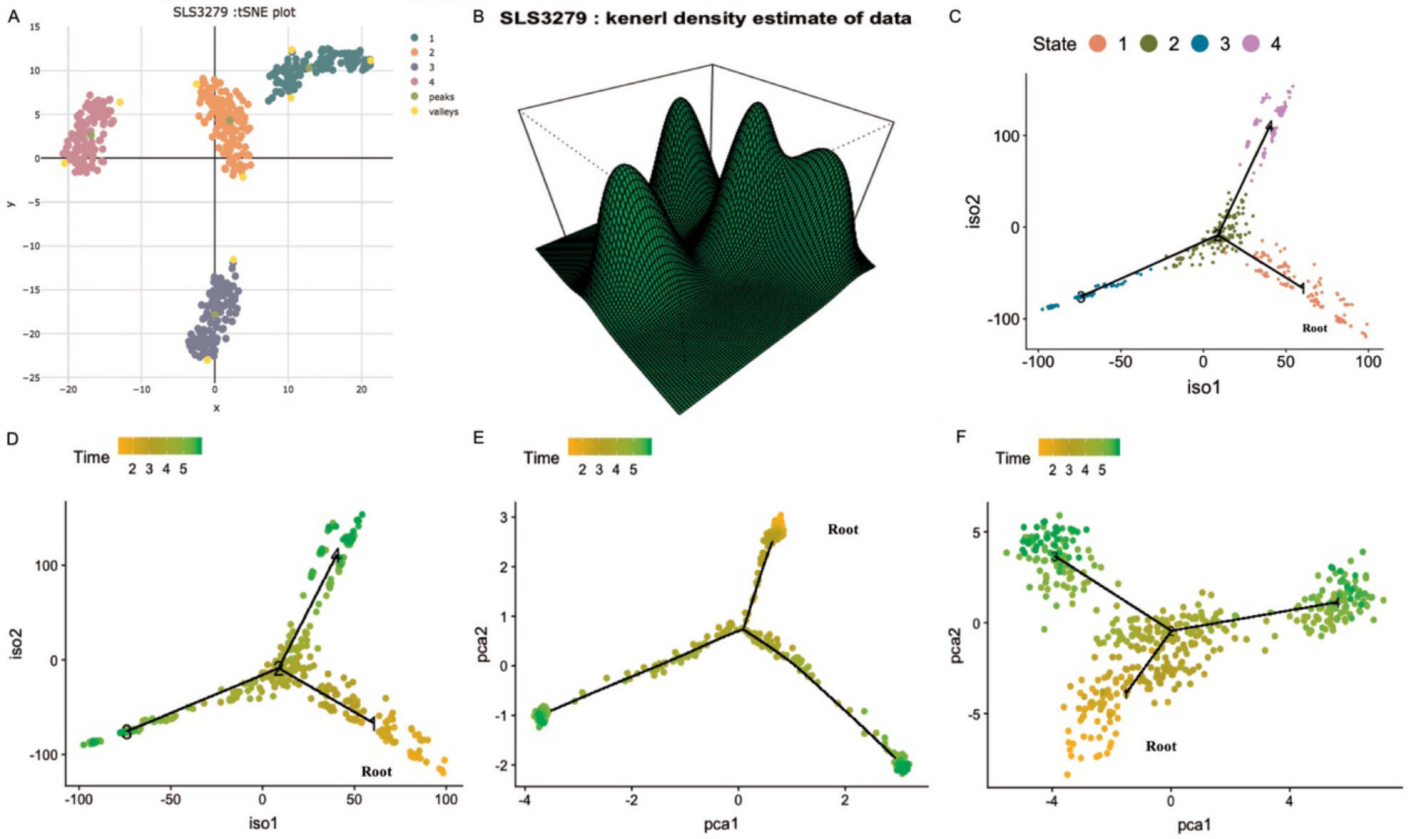
SLS3279 results. (A) The cell clusters, peaks, and valleys in the t-SNE embedding. (B) The cell density landscape. (C) The cell trajectory in the isometric embedding. (D) The cell trajectory reconstructed by LISA. (E) The cell trajectory reconstructed by Monocle2. (F) The cell trajectory reconstructed by TSCAN. In (D)-(F), the true time labels are shown.

First, we used the simulated dataset to verify the capability of LISA. In the simulated dataset, it contains two cell lineages. We did PCA for SLS3279, and all PCs were retained. The PCA result was input for t-SNE. Fig. 2A shows the cell clusters, peaks, and valleys that were derived from the t-SNE embedding by kNN-graph and hierarchical clustering described in the Methods section. The cell densities were shown in Fig. 2B. Correspondingly, it contains four peaks. Then we performed Landmark Isomap and built the MST of the cluster centers (Fig. 2C). We obtained the cell trajectory with two terminal lineages by setting cluster 1 as the root cluster (Fig.2D).

For comparison, we applied Monocle2 and TSCAN to the simulated datasets. In the Monocle2 result, the cells were more concentrated at the ends of the branches (Fig. 2E). And the Spearman correlation coefficients between the estimated pseudo-time and true time labels were higher in LISA (0.97) than in Monocle2 (0.92). In the TSCAN result, the cells were more dispersed (Fig. 2F) and the global pseudo-time was not obtained. These results showed the potential of LISA in reconstructing cell trajectory and pseudo-time.

### 3.3. Application to the EMTAB dataset

**Figure 3.**
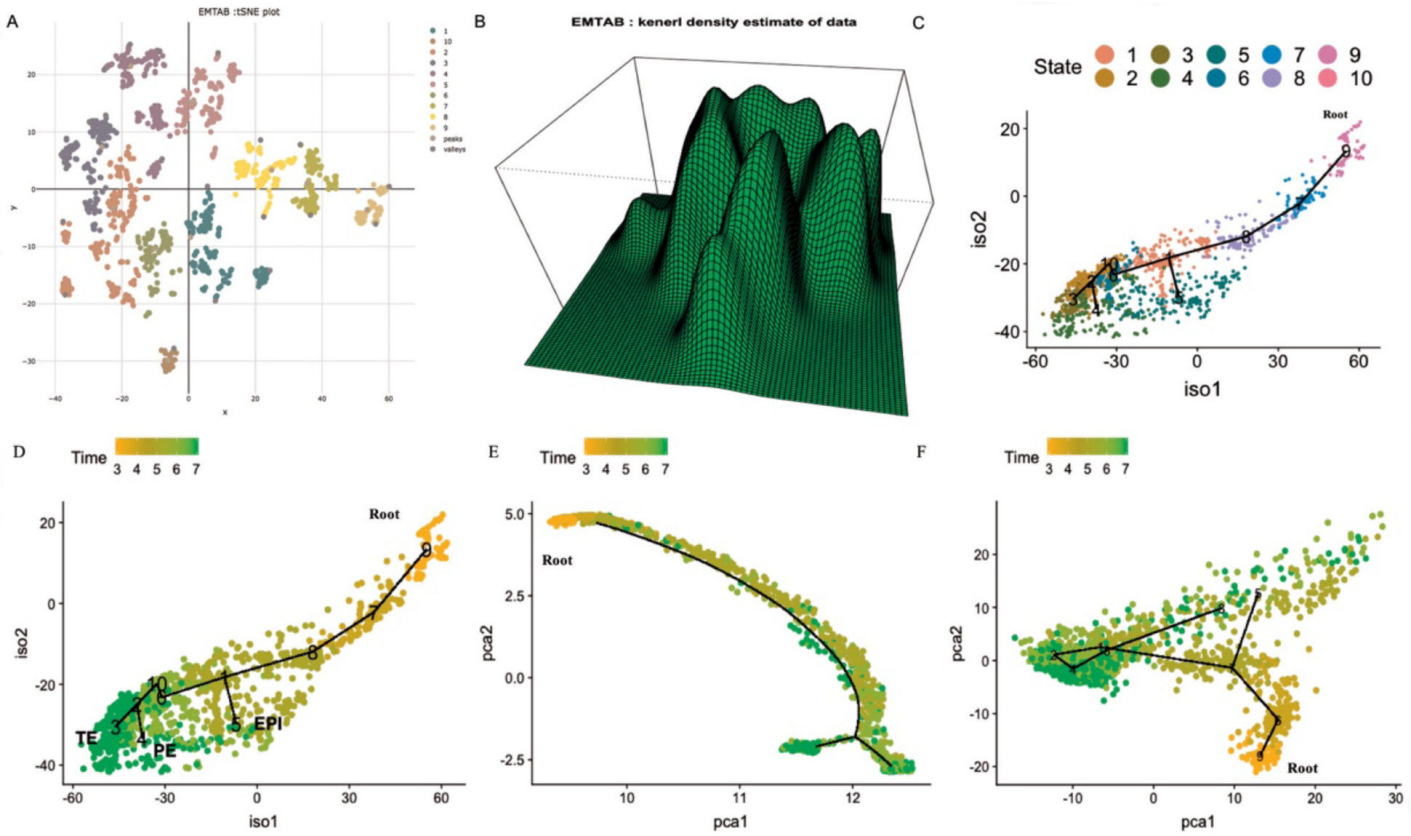
EMTAB results. (A) The cell clusters, peaks, and valleys in the t-SNE embedding. (B) The cell density landscape. (C) The cell trajectory in the isometric embedding. (D)The cell trajectory reconstructed by LISA. (E) The cell trajectory reconstructed by Monocle2. (F) The cell trajectory reconstructed by TSCAN. In (D)-(F), the true time labels are shown.

**Figure S1.**
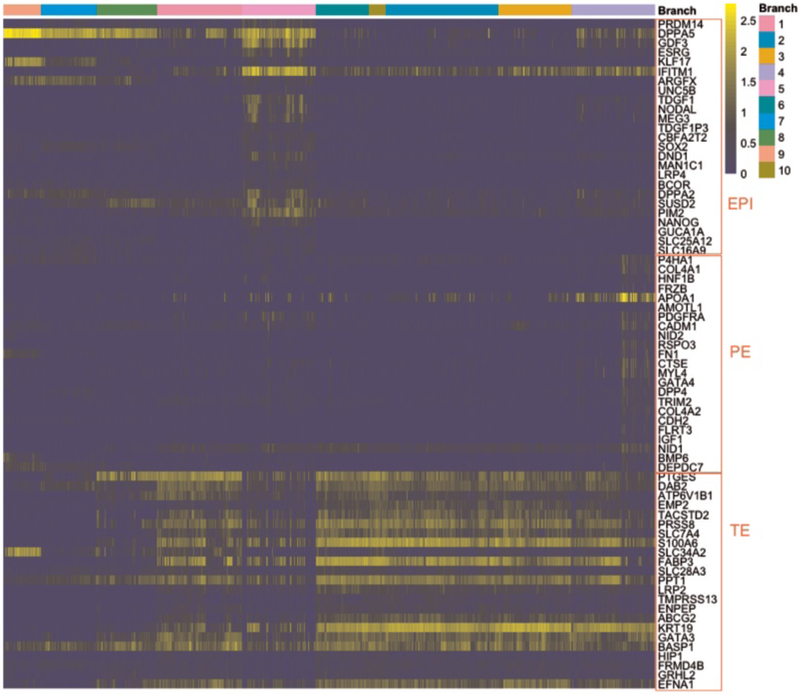
The gene expression heatmap of marker genes from three cell types (EPI, PE, TE). The branch names correspond to the cell clustering results.

We applied LISA to the EMTAB dataset which contains 1,364 cells [15]. It includes human preimplantation embryos cells developed into epiblast (EPI), primitive endoderm (PE) and trophectoderm (TE) cells from E3 to E7. The cell clusters, peaks and valleys were shown in Fig. 3A. The cell density plot was shown in Fig. 3B implying the complexity of cell clustering. We obtained 10 cell clusters. We then built the main cell trajectory in the isometric embedding (Fig. 3C). By setting cluster 9 as the root of cell trajectory, it clearly shows three terminal lineages in the cell differentiation path leading to cluster 5, 4, and 3, respectively. To understand the nature of the cell lineages, we used the 71 maker genes from EPI, PE and TE [15] to examine the genes expression patterns in different cell clusters (Fig. S1 in Appendix). It can be seen that cluster 5 is enriched for EPI marker genes, cluster 4 is enriched for PE marker genes, and cluster 3 is enriched for TE marker genes.

As comparison, applying Monocle2 to the EMTAB dataset resulted in only two terminal lineages (Fig. 3E). Moreover, the Spearman correlation coefficients between the estimated pseudo-time and true time points were much higher in LISA (0.90) than in Monocle2 (0.77). The cell trajectory from TSCAN were shown in Fig. 3F, which also contains only two lineages.

### 3.4. Application to the Zebrafish dataset

We further applied LISA to a large zebrafish embryo differentiation dataset which contains 38,731 cells [2]. There are mainly three cell lineages including axial mesoderm, other mesendoderm, and ectoderm. In addition, it contains primordial germ and enveloping layer cells.

**Figure 4.**
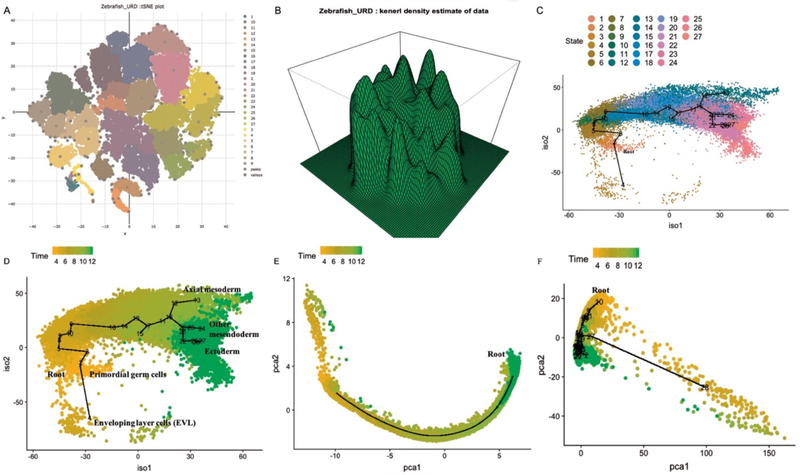
Zebrafish results. (A) The cell clusters, peaks, and valleys in the t-SNE embedding. (B) The cell density landscape. (C) The main cell trajectory in the isometric embedding. (D)The cell trajectory reconstructed by LISA. (E) The cell trajectory reconstructed by Monocle2. (F) The cell trajectory reconstructed by TSCAN. In (D)-(F), the true time labels are shown.

**Figure S2.**
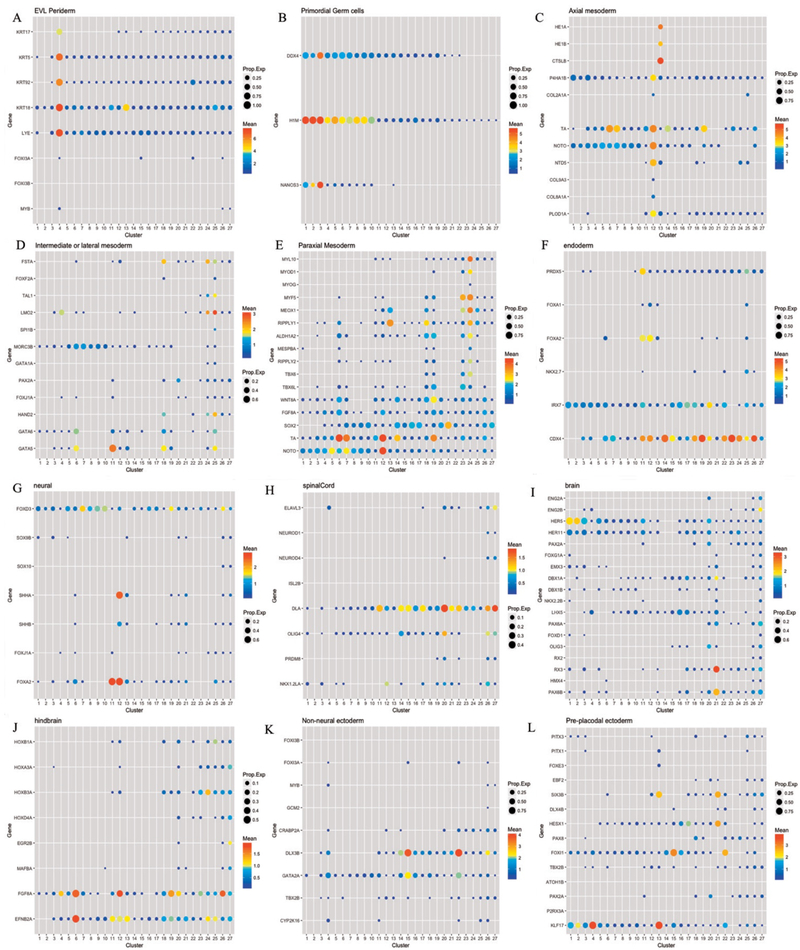
The expression patterns of the marker genes of the 12 cell types in 27 clusters. (A) Enveloping layer cell and Periderm. (B) Primodial germ. (C) Axial mesoderm. (D) Intermediate or lateral mesoderm. (E) Paraxial Mesoderm. (F) Endoderm. (G) Neuron. (H) Spinal cord. (I) Brain. (J) Hindbrain. (K) Non-neural ectoderm. (L) Pre-placodal ectoderm.

The cell clusters, peaks, and valleys of the zebrafish dataset is shown in Fig. 4A. The cell density plot is shown in Fig. 4B. We identified 27 cell clusters, peaks and valleys. We used the cell type marker genes [2] to investigate whether the main cell trajectories (Fig. 4C) are corresponding to known paths. As shown in Fig. S2 in Appendix, the endoderm marker genes were enriched in cluster 11 and 12. The primordial germ cell markers were enriched in cluster 1, 2 and 3. The enveloping layer cells (EVL) marker genes were enriched in cluster 4. The intermediate/lateral mesoderm marker genes were enriched in cluster 18, 24 and 25. The axial mesoderm marker genes were enriched in cluster 12 and 13. The paraxial mesoderm marker genes were enriched in cluster 19, 24 and 26. The pre-placodal ectoderm marker genes were enriched in cluster 21, 22, 26 and 27. The non-neural ectoderm marker genes were enriched in cluster 22, 23, 25, and 27. The hindbrain, fore/mid brain, neural crest and spinal cord marker genes were enriched in cluster 26 and 27. Based on the gene expression patterns, the cell lineage along cluster 11, 18, 12, and 13 was mainly corresponding to endoderm and axial mesoderm. The lineage along cluster 18, 20, 23, and 24 was mainly corresponding to intermediate/lateral mesoderm and paraxial mesoderm. The lineage along cluster 20, 21, 22, 25, 26, and 27 was corresponding to ectoderm which includes pre-placodal ectoderm, non-neural ectoderm, hindbrain, fore/mid brain, neural crest, and spinal cord. The lineage along cluster 1 was mainly corresponding to primordial germ cells. The lineage along cluster 4 was corresponding to EVL. Overall, the main cell trajectories reconstructed by LISA were consistent with those in Farrell *et al.* [2]. We set cluster 1 as the root of cell trajectory and estimated the pseudo-time of all cells. The Spearman correlation coefficients between the true time labels and the pseudo time reconstructed by LISA is 0.91.

As comparison, Monocle2 only generated one cell lineage (Fig. 4E). Furthermore, the pseudo-time reconstructed by Monocle2 is reverse to the true time labels resulting in a negative Spearman correlation coefficient. The TSCAN derived cell lineages were compressed and hard to be distinguished (Fig. 4F). Also, the cell lineages were not corresponding to Farrell *et al.* [2].

### 3.5. Performance comparisons

**Figure 5.**
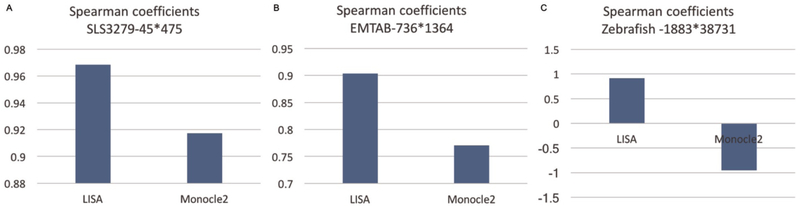
Comparing the Spearman correlation coefficients between the pseudo-time and the true time labels for different datasets using Monocle 2 and LISA. (A) SLS3279. (B) EMTAB. (C) Zebrafish.

To estimate the pseudo-time of all cells, we set the root cluster based on the initial time point. In our comparisons, the clusters which contain the most numbers of cells at the initial time point were selected as the roots for both LISA and Monocle2. However, in the Zebrafish dataset, Monocle2 only showed one lineage. In this case, the root cell was determined by Monocle2 automatically. The pseudo-time reconstructed by LISA was more consistent with the true time points than Monocle2 did (Fig. 5).

**Figure 6.**
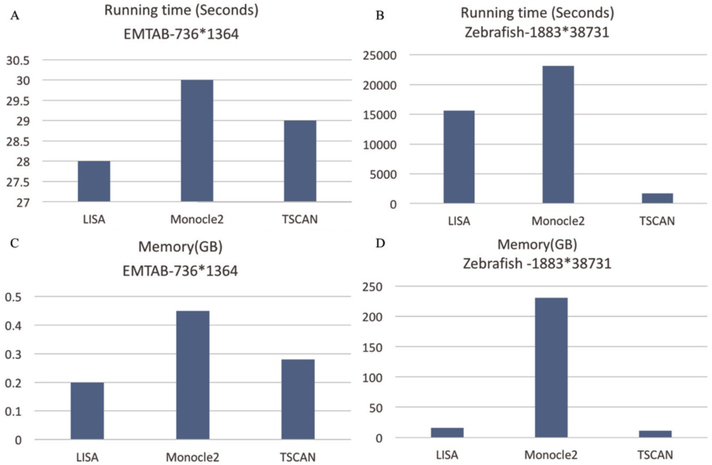
Computation time and memory usage of EMTAB and Zebrafish using LISA, Monocle2 and TSCAN. (A) The computation time on the EMTAB dataset. (B) The computation time on the Zebrafish dataset. (C) The memory usage of the EMTAB dataset. (D) The memory usage of the Zebrafish dataset.

Overall, LISA showed better performance on reconstructing cell trajectory than Monocle2 and TSCAN did. Moreover, LISA used lower amount of computation time and required dramatically less memory than Monocle2 did (Fig. 6A–D). LISA used lower amount of computation time and memory than TSCAN did on the EMTAB dataset (Fig. 6A and C), and more computation time and similar memory usage compared to TSCAN on the Zebrafish dataset (Fig. 6B and D). In addition, in our tests, as cell number increased to exceed 50,000, Monocle2 was not able to estimate the pseudo-time, and TSCAN was not able to run its clustering procedure.

## 4. Discussion

LISA is a new tool to reconstruct cell trajectory and pseudo-time of cells from scRNA-seq data. It uses kNN-graph and hierarchal clustering for identifying cell clusters, peaks, and valleys in the t- SNE embedding in an unsupervised way. It then uses the fast Landmark Isomap to derive the global geometrical structure of the data to estimate the main cell trajectory. Finally, it projects individual cells on the main cell trajectory and computes the global pseudo-time.

The assessments of cell trajectory and global pseduo-time reconstruction of LISA demonstrate its improved performance over existing methods such as Monocle2 and TSCAN. Meanwhile, LISA runs faster and requires less memory usage than Monocle2 does. In LISA, the root cluster can be set by the users for customized cell trajectory and pseudo-time analysis. Existing biological knowledge of specific gene sets, e.g., known marker genes of cell types or states, can be used to reveal the biological meanings of the reconstructed cell lineages. In summary, LISA is an accurate, efficient, and flexible tool that can be broadly applied to massive scRNA-seq datasets.
